# Open-Air Treatment in London

**Published:** 1901-04-13

**Authors:** 


### OPEN-AIR TREATMENT IN LONDON.

The *St. Thomas's Hospital Gazette* gives details of two cases and mentions a third which have been undergoing the "open-air treatment" on the balconies of the hospital during the past winter. In all the cases there has been improvement, and in the general condition great improvement. In the first case (under Dr. Mackenzie) there was dulness over the front of the right side of the chest, impaired resonance in the upper part of the axilla, and cavity sounds. In the left lung no physical signs of disease were detected. He was coughing up a quantity of greenish sputum, tinged with blood, and his breath was very offensive. While in the ward he several times brought up large quantities of blood, his appetite was bad, he was losing flesh, and was gradually becoming worse. On August 6th his bed was put out on the balcony, and since then he has lived entirely out of doors, only coming into the ward to dress or to be examined. During the first month there was little or no improvement; his temperature kept up, there was hæmoptysis, and his general condition was such as to afford very little hope of recovery. After about a month, however, he began to improve. There has been no hæmoptysis since September 14th, the sputum has diminished in quantity and become less offensive, he has gained two stones in weight, is looking well, his appetite is good, and he is able to take a fair amount of exercise without fatigue. The patients have been protected from wind by tarpauline screens, which are put up on windy days according to the direction of the wind and rain, the beds being covered, if necessary, by mackintoshes. Otherwise the beds are quite exposed on the balcony. Warmth is secured by plenty of blankets, the use of gloves and cap, and by hot-water bottles if required. The first patient has been out all the winter, during dense fogs, heavy snow, frost, wet and stormy nights; but in spite of these conditions he much prefers sleeping outside to coming into the ward. Of course he has been induced to take as much food as he could without upsetting his stomach.

The second patient (under Dr. Hawkins) dated his illness from about eighteen months before his admission. He had had constant cough and expectoration, but no hæmoptysis. He had night sweats with loss of appetite and weight. There were physical signs of phthisis in both apices; worse on the right, where the breath sounds were almost cavernous. This patient, however, began to improve in the ward before he began the out-door treatment. This he commenced on January 2nd. He is now much better, the physical signs are less marked, the cough and expectoration have very greatly diminished, and he looks a "new man." He is fat and rosy, and his only trouble is the "stuffiness" of the ward when he comes in to be examined. As to the third case (also under Dr. Hawkins), it is too soon to say much, but her weight is increasing, and there is already a remarkable increase in her blood corpuscles, although not a corresponding one in the percentage of hæmoglobin. Such cases as these seem to us even more important than cases of cure in the Black Forest, or at mountain health resorts, or in other places where the climatic influences to which the patients are exposed are so obviously an improvement upon those of home that it becomes difficult to say how much is due to them and how much to the open air. But when we find patients lying out of doors all day and all night, and getting well in the midst of their accustomed London atmosphere, and this not merely on the breezy heights of Hampstead, as at the North London Consumption Hospital, but also down amid the river fogs at St. Thomas's Hospital, we seem to see that the benefit really comes from the outdoor life, or, to put the matter in another and perhaps a truer way, from the removal of those deleterious influences which we have not yet learned to eliminate from our indoor life.

